# Machine Learning-Assisted Optimization of Drug Combinations in Zeolite-Based Delivery Systems for Melanoma Therapy

**DOI:** 10.1021/acsami.3c18224

**Published:** 2024-01-25

**Authors:** Ana Raquel Bertão, Filipe Teixeira, Viktoriya Ivasiv, Pier Parpot, Cristina Almeida-Aguiar, António M. Fonseca, Manuel Bañobre-López, Fátima Baltazar, Isabel C. Neves

**Affiliations:** † CQUM, Centre of Chemistry, University of Minho, Campus de Gualtar, 4710-057 Braga, Portugal; ‡ Life and Health Sciences Research Institute (ICVS), School of Medicine, University of Minho, 4710-057 Braga, Portugal; § ICVS/3B’s - PT Government Associate Laboratory, University of Minho, 4710-057 Braga/Guimarães, Portugal; ∥ Advanced (Magnetic) Theranostic Nanostructures Lab, Nanomedicine Group, International Iberian Nanotechnology Laboratory (INL), Av. Mestre José Veiga, 4715-330 Braga, Portugal; ⊥ CEB - Centre of Biological Engineering, University of Minho, 4710-057 Braga, Portugal; # CBMA - Centre of Molecular and Environmental Biology, University of Minho, 4710-057 Braga, Portugal

**Keywords:** zeolite, ZDS formulations, machine learning, ANN models, microbial infections, melanoma therapy

## Abstract

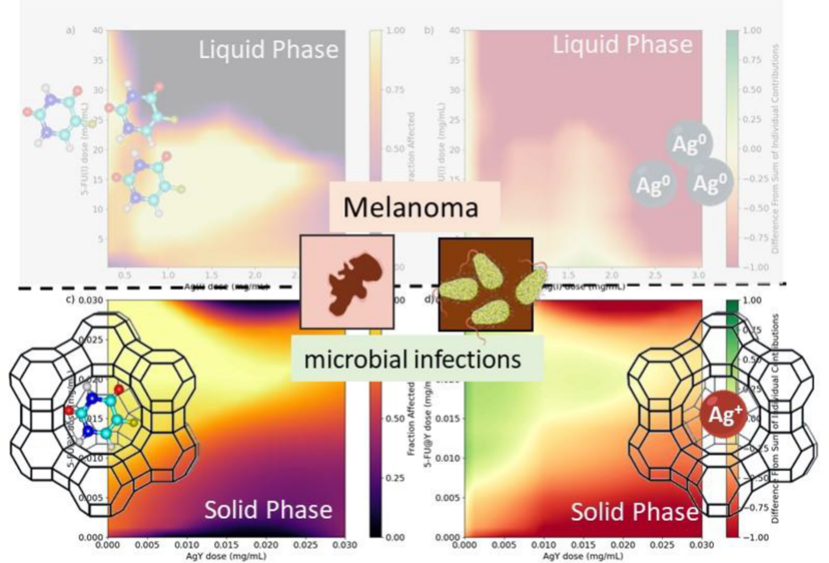

Two independent artificial neural network (ANN) models were used to determine the optimal drug combination of zeolite-based delivery systems (ZDS) for cancer therapy. The systems were based on the NaY zeolite using silver (Ag^+^) and 5-fluorouracil (5-FU) as antimicrobial and antineoplastic agents. Different ZDS samples were prepared, and their characterization indicates the successful incorporation of both pharmacologically active species without any relevant changes to the zeolite structure. Silver acts as a counterion of the negative framework, and 5-FU retains its molecular integrity. The data from the A375 cell viability assays, involving ZDS samples (solid phase), 5-FU, and Ag^+^ aqueous solutions (liquid phase), were used to train two independent machine learning (ML) models. Both models exhibited a high level of accuracy in predicting the experimental cell viability results, allowing the development of a novel protocol for virtual cell viability assays. The findings suggest that the incorporation of both Ag and 5-FU into the zeolite structure significantly potentiates their anticancer activity when compared to that of the liquid phase. Additionally, two optimal AgY/5-FU@Y ratios were proposed to achieve the best cell viability outcomes. The ZDS also exhibited significant efficacy against *Escherichia coli* (*E. coli*) and *Staphylococcus aureus* (*S. aureus*); the predicted combination ratio is also effective against *S. aureus*, underscoring the potential of this approach as a therapeutic option for cancer-associated bacterial infections.

## Introduction

Cancer is expected to be the leading cause of morbidity and death worldwide in the 21st century, with approximately 19.3 million new cases and almost 10.0 million cancer deaths occurring in 2020.^1^ By 2040, the worldwide number of cancer cases is predicted to reach 28.4 million, which represents a 47% increase from the levels recorded in 2020.^1^ Alongside alarming statistics, the increasing complexity of cancer poses another growing challenge in terms of treatment options. Recently, in the work of Hanahan,^2^ new “emerging hallmarks” and “enabling characteristics”, associated with the core hallmarks of cancer, were mentioned. Amidst the ever-increasing pool of published evidence exploring novel facets of the disease, the compelling data regarding the impact of polymorphic variability of microbiomes on cancer phenotypes grow stronger.^2^ Recent findings indicate that malignant tumors exhibit distinct bacterial profiles, and some of these bacteria may potentially undermine the effectiveness of chemotherapy approaches.^3−5^ In combination with other forms of drug resistance, this emerging evidence stresses one of the primary drawbacks of monochemotherapy (the use of a single antineoplastic agent), despite being the standard and most prevalent therapeutic approach presently.^6^ Combining drugs has been acknowledged as a viable approach to address resistance, reduce the chances of recurrence, minimize dosages, and enhance the potential for drug repurposing.^6,7^

The use of biocompatible materials as carriers for various anticancer agents provides several benefits, such as the potential for controlled and targeted release of both agents and reduced toxicity for healthy cells.^8^ Different formulations can be designed based on the specific application scenario in which they will be utilized. To the best of our knowledge, the utilization of zeolites as hosts to investigate their combined anticancer potential has not been reported previously. Furthermore, the investigation and determination of the formulation and ratio that optimize their combined efficiency in this context are of utmost importance. In that regard, machine learning (ML) models are gaining popularity as valuable tools to explore potential drug combinations.^9^

In the case of zeolites, ML approaches—specifically artificial neural networks (ANN)—are currently being used to forecast chemical reaction pathways for their synthesis and various applications.^10−12^ The prediction of the presence and crystallinity of beta zeolites and competing phases during the synthesis, using experimental parameters, was possible with the use of ANN.^13^ An ANN model was also utilized to anticipate the adsorption process of tetracycline from aqueous solutions using zeolites.^14^ To the best of our knowledge, the use of ANN was never employed to predict dose–response curves. Usually, in this case other algorithms have already been used. For example, the optimal discriminant analysis (ODA) machine-learning algorithm was used to analyze the data from a study measuring the responses of the blood flow in the forearm to the intra-arterial administration of isoproterenol. The authors showed that ODA should be considered the primary analytic approach in dose–response applications.^15^

Having this in mind, several zeolite-based delivery systems (ZDS) with silver (Ag, antimicrobial, and anticancer agents) and 5-fluorouracil (5-FU, classical antineoplastic agent) were prepared. Both Ag- and 5-FU-containing zeolites have already been described in the literature for anticancer and antimicrobial applications.^16−18^ This study involved testing various ZDS-based formulations to identify the most effective antitumor response. The resulting data were analyzed using ANN. Skin cancer (melanoma) was chosen as the cancer model for this study, given its substantial economic burden on healthcare services arising from its increasing incidence over the past decades.^1,19,20^ Furthermore, the literature extensively addresses the abundant presence of bacteria on the skin and its potential contribution to the development of skin cancer through infections.^21−23^ Moreover, individuals with compromised immune systems are at increased risk of developing secondary infections.^22^ In this context, preliminary studies were also conducted with two bacterial species, *E. coli* and *S. aureus*, to explore the potential of these ZDS samples for addressing cancer-related bacterial infections. The best ratio combinations predicted by ANN models were also studied with these bacteria.

## Experimental Section

### Preparation and Characterization of the Zeolite Delivery Systems (ZDS)

Several delivery systems based on zeolites (ZDS) were prepared to study their potential as antitumor agents in a melanoma cell model. For that the host zeolite NaY (CBV100, Zeolyst International) was used as support for silver (Ag^+^) and 5-fluorouracil (5-FU) as antimicrobial and antineoplastic agents, respectively. The ZDS sample with silver ions (AgNO_3_, Fisher Scientific) was prepared with NaY (AgY). The ZDS sample with Ag was prepared by the ion exchange method described in ref (24). Aluminum foil was employed to cover the volumetric flask utilized in the reaction, to prevent the unwanted reduction of Ag^+^ ions due to silver sensibility to light exposure.^25^ The suspensions were maintained under constant stirring at 300 rpm for 24 h at room temperature, filtered off, washed with deionized water, and dried overnight at 60 °C. Finally, the resulting powder was calcined at 350 °C for 4 h.

Other ZDS were obtained by encapsulation of 5-fluoro-1*H*,3*H*-pyrimidine-2,4-dione (5-fluorouracil, 5-FU, Sigma-Aldrich) into the AgY sample (Ag(5-FU)@Y) and NaY ((5-FU)@Y). The encapsulation of 5-FU was performed by the liquid adsorption method, following a previously employed procedure.^26^ Before 5-FU loading, the AgY powder was pretreated at 150 °C for 4 h to avoid the presence of water molecules inside the pores. The loading of the drug into the zeolite structure was achieved by adding 200 mg of AgY zeolite powder to 25 mL of a 5-FU (0.577 mmol) solution in 80% acetone/20% water (v/v). The mixture was kept under constant stirring at room temperature for 48 h and sealed to prevent solvent evaporation. After this period, the resulting mixture was filtered and washed once with the same solvent to remove the nonencapsulated 5-FU and dried in an oven at 60 °C for 12 h. The (5-FU)@Y sample was prepared with an initial solution of 5-FU (0.999 mmol) using the same experimental conditions.

For the experimental design, several proportions of AgY/(5-FU)@Y or AgY/Ag(5-FU)@Y were formulated with ZDS sample mass ratios of 1:1, 1:2, 1:5, and 5:1. A stock solution (1 mg/mL) was prepared with ZDS samples using the mentioned mass ratios at RT.

The X-ray photoelectron spectroscopy (XPS) surface measurements of the ZDS samples were conducted using an ESCALAB 250XI (Thermo Fisher Scientific) with Al Kα X-rays (1486.6 eV). Measurements were performed with a 650 μm spot size at a base pressure lower than 10^–10^ mbar. The obtained XPS data were analyzed using Thermo Scientific Avantage software.

The loading of 5-FU and the thermal stability of the samples were determined by thermogravimetric analysis (TGA) in an STA 409 PC Luxx Netzsch thermal analyzer. The atmosphere used was high-purity air (99.99% minimum purity) with a constant flow rate of 50 cm^3^/min. Crucibles of alumina oxide, supplied by Netzsch, were used to hold a certain amount of the samples and were heated for 65 min, between 50 and 700 °C at a heating speed of 10 °C/min.

The morphology and size of ZDS were assessed using transmission electron microscopy (TEM). A JEOL JEM-2100 HT instrument with an accelerating voltage of 200 keV was employed for this purpose. The TEM micrographs were acquired at different magnifications using the OneView 4k × 4k charge-coupled device (CCD) camera.

The silver amount in the samples was determined by inductive coupled plasma (ICP) on ICP-AES Horiba Jobin Yvon model Ultima equipment according to the SMEWW 3120 method.

### Release Studies *In Vitro*

To conduct the *in vitro* release study of 5-FU, 10 mg of Ag(5-FU)@Y was added to 50 mL of solution of phosphate-buffered saline (PBS, Sigma-Aldrich). This solution was designed to mimic body fluid, with a pH of 7.4, and the temperature was maintained at 37 °C. At predetermined intervals, 1 mL aliquots were withdrawn from the mixture and immediately replaced with an equal volume of fresh buffer solution to ensure a constant released medium volume. The release study was performed over 6 h. The collected aliquots were centrifuged and filtered using disposable filter devices with a 0.20 μm pore nylon membrane. The absorbance value at λ = 266 nm was recorded for each withdrawn sample with a UV-2501PC spectrophotometer (Shimadzu). PBS was used as the blank sample to adjust the baseline. The amount of 5-FU released was determined based on the methodology described in ref (27).

### Cytotoxicity Assays

#### Melanoma Cells

The A375 melanoma cell line, obtained from the American Type Culture Collection (ATCC, USA), was cultured routinely in supplemented Dulbecco’s Modified Eagle’s Medium (DMEM, Gibco, Invitrogen) from Gibco, Invitrogen. The culture medium was supplemented with 10% (v/v) heat-inactivated fetal bovine serum (FBS Gibco, Invitrogen) and 1% (v/v) penicillin–streptomycin (Pen/Strep, Gibco, Invitrogen). The cells were maintained at 37 °C in a 5% CO_2_ humidified atmosphere.

To evaluate the effect of the ZDS samples, either alone or in combination at fixed ratios, on cell viability *in vitro*, the Sulforhodamine B (SRB) colorimetric assay was conducted, as previously described.^28^ For this experiment, A375 cells were seeded in triplicate in 96-well culture plates at a density of 10 × 10^3^ cells per well. The plates were incubated overnight at 37 °C under a 5% CO_2_ atmosphere. The medium was then replaced with sequential dilutions of a stock sample suspension (0.5 mg/mL). To ensure better homogenization, each stock suspension (0.5 mg/mL) was sonicated in an ultrasonic bath for 3 min before use. The cells were further incubated and changes in cell viability were monitored for 72 h. After the incubation period, the medium was removed from all wells, and the cells were fixed by adding 50 μL of 10% (w/v) trichloroacetic acid (TCA) and stored at 4 °C for 1 h. Subsequently, TCA was discarded, and the wells were washed four times with deionized water, being left to dry at room temperature. The cells were then stained with 50 μL of SRB solution for 30 min at room temperature. After removing the SRB solution, the cells were washed four times with 1% (v/v) acetic acid and allowed to dry. The protein-bound dye was solubilized by adding 100 μL of 10 mM Tris base solution per well. Finally, the optical density (OD) was measured at 530 nm using a microplate reader, Synergy Biotek H1, coupled with BioTek Gen5 software. The results were presented as the percentage of viable cells compared to the control condition, which was assumed to have 100% cell viability.

#### Bacterial Cells

The antimicrobial activity of AgY was evaluated by using some bacterial strains as predictive models. *Staphylococcus aureus* (ATCC 6538) and *Escherichia coli* (CECT 423)—obtained from the culture collection of the Biology Department at the University of Minho—were inoculated into 10 mL of sterile Luria–Bertani broth (LB) and incubated at 37 °C and 200 rpm until OD at 600 nm reached 0.6–0.8. These stock bacterial suspensions were diluted to 1.0 × 10^7^ cells/mL before use.

The antibacterial activity was evaluated by an adaptation of the resazurin-based turbidimetric assay.^29^ The resazurin solution was prepared by dissolving 6 mg of resazurin powder in 50 mL of sterile distilled water, stored at 4 °C, and protected from light. In a 96-well round-bottom microtiter plate, AgY and NaY mixed with LB medium were serially diluted from 4 to 0.125 mg/mL. 10 μL of a stock bacterial suspension was added to each well, resulting in a final concentration of 10^6^ cells/mL and a final volume of 100 μL per well. Control samples were included on each plate covering various columns: LB medium only, individual bacterial suspensions, and ZDS samples without bacteria. The plates were prepared in triplicate and incubated at 37 °C for 24 and 48 h, followed by the addition of 10 μL of resazurin solution to each well and further incubation for 2 h at 37 °C in the dark. The color change was then visually evaluated. The presence of viable cells (indicating growth) was expressed by a color transition from blue to pink. The minimum inhibitory concentration (MIC) value was expressed as the lowest concentration of AgY that prevented a color change of resazurin.

To test the best-predicted ratios of AgY/(5-FU)@Y by ANN models, an agar well diffusion test was performed with *E. coli* and *S. aureus* to evaluate the bacterial growth inhibition in the presence of 50 μg/mL of the ratios (5-FU)@Y and NaY. Each bacterial inoculum, prepared as described earlier, was applied to a sterile cotton swab. The swab was then used to gently wipe the surface of a LBA plate. Following that, 50 μL of each ratio sample at a concentration of 50 μg/mL was added to the previously formed wells on the plate. A commercial disc containing the antibiotics amoxicillin/clavulanic acid (Sensi-Disk amoxicillin/clavulanic acid 20/10 μg, Fisher Scientific) served as the positive control. As a negative control, two LBA plates were prepared with each bacterium, but the wells remained empty. After an incubation period of 24 h at 37 °C, the plates were examined for the presence of growth inhibition zones.

### Machine Learning (ML) Approaches

#### Experimental Design: Full Factorial Design

To obtain experimental data for the cytotoxicity cell viability assays, various combinations of ZDS samples were used. A preliminary assessment was conducted using a 3^*k*^ factorial design, where *k* represents two variables (the Ag/5-FU ratio and the concentration), to assess the correlation between them. The mixtures of the ZDS samples were performed by the combination of Ag(5-FU)@Y and (5-FU)@Y, or AgY and (5-FU)@Y (solid phase). All experiments were performed in triplicate. A similar procedure was performed with nitrate silver solutions (AgNO_3_) and 5-FU solutions, considering the available concentration of both species in the ZDS samples (liquid phase).

#### Virtual Cell Viability Assays

Two independent artificial neural network (ANN) models were developed to target cell viability under the assay conditions described above. The first model (model A) targeted cell viability as a function of the mass concentration of 5-FU and Ag in the aqueous combinations Ag(aq) and 5-FU(aq) in the liquid phase, while the second model (model B) targeted cell viability as a function of the mass concentrations of the ZDS samples, AgY, 5-FU@Y, and Ag(5-FU)@Y in the zeolite-based compositions tested the solid phase. In both cases, the cell viability determined in each assay was used, instead of the average value per mass concentration or ZDS load composition, yielding 236 data points describing the aqueous combinations and 123 points describing the cell viability against different ZDS compositions (Tables S1 and S2, respectively). Both models share the same architecture, implemented using the Scikit-learn package, version 1.1.3.^30^

Each model consists of a three-stage data processing pipeline: in the first stage, data were standardized (subtraction of the mean value and scaling to unit variance on each feature), and then a new feature matrix was generated consisting of all polynomial combinations of the features with degree up to *n*_poly_. This stage is finally followed by the ANN algorithm (multilayer perceptron regressor), the topology of which was limited to a single hidden layer with *N*_n_ neurons considering the amount of available data. In both cases, a 60:40 split between the training and validation data was carried out. The hyperparameters *n*_poly_, *N*_n__,_ and the learning rate of the ANN stage (α) were optimized using a standard 5-fold cross-validation protocol in which the train data was further divided into five subsets. Four of them were used to train a probe model with a given combination of these hyperparameters. The fifth subset was used to test the model’s ability to predict new data. By rotating the subset used for the testing phase, we were able to obtain five probe models for each combination of *n*_poly_, *N*_n_, and α and selected the hyperparameter combination yielding the highest *r*^2^ in the test phase (averaged by the five models sharing the same hyperparameters). This optimization routine took place in two rounds: in the first round, *N*_n_ varied between 10 and 100 in increments of 10, with 50 being the most promising value. Then, a second round was performed scanning values of *N*_n_ between 41 and 59, which confirmed *N*_n_ = 50 as the best value for this hyperparameter. In all cross-validation studies, *n*_poly_ varied between 1 and 6, and α varied between 10^–5^ and 10^–2^ in a logarithmical fashion; the optimized values found during the cross-validation studies were 2 and 1 × 10^–3^, respectively. All results from these cross-validation routines are provided in the Supporting Information. At the end of the hyperparameter optimization protocol, each model was trained with the optimized hyperparameters and the full train set.

Both models were tested by comparing their predictions for the validation set with the experimental data available for those assays. Further exploration of the model’s response was performed by scanning the model’s response over a systematic grid of concentration values for Ag(aq) and 5-FU(aq) for model A (liquid phase) and AgY, 5-FU@Y, and Ag(5-FU)@Y for model B (solid phase). The data from these virtual assays allowed dose–response parameters to be estimated for each of the intervening species by fitting the logistic dose–response curve (eq 1):
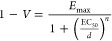
1where *V* is the cell viability (as a fraction of the total population) predicted by the model, *E*_max_ is the maximum effect predicted for that component, EC_50_ is the dose needed to achieve 50% of *E*_max_, and *n* is the Hill coefficient. The Python notebooks used for all data analysis are provided in the Supporting Information.

## Results and Discussion

### Zeolite-Based Delivery Systems (ZDS)

**Figure 1:**
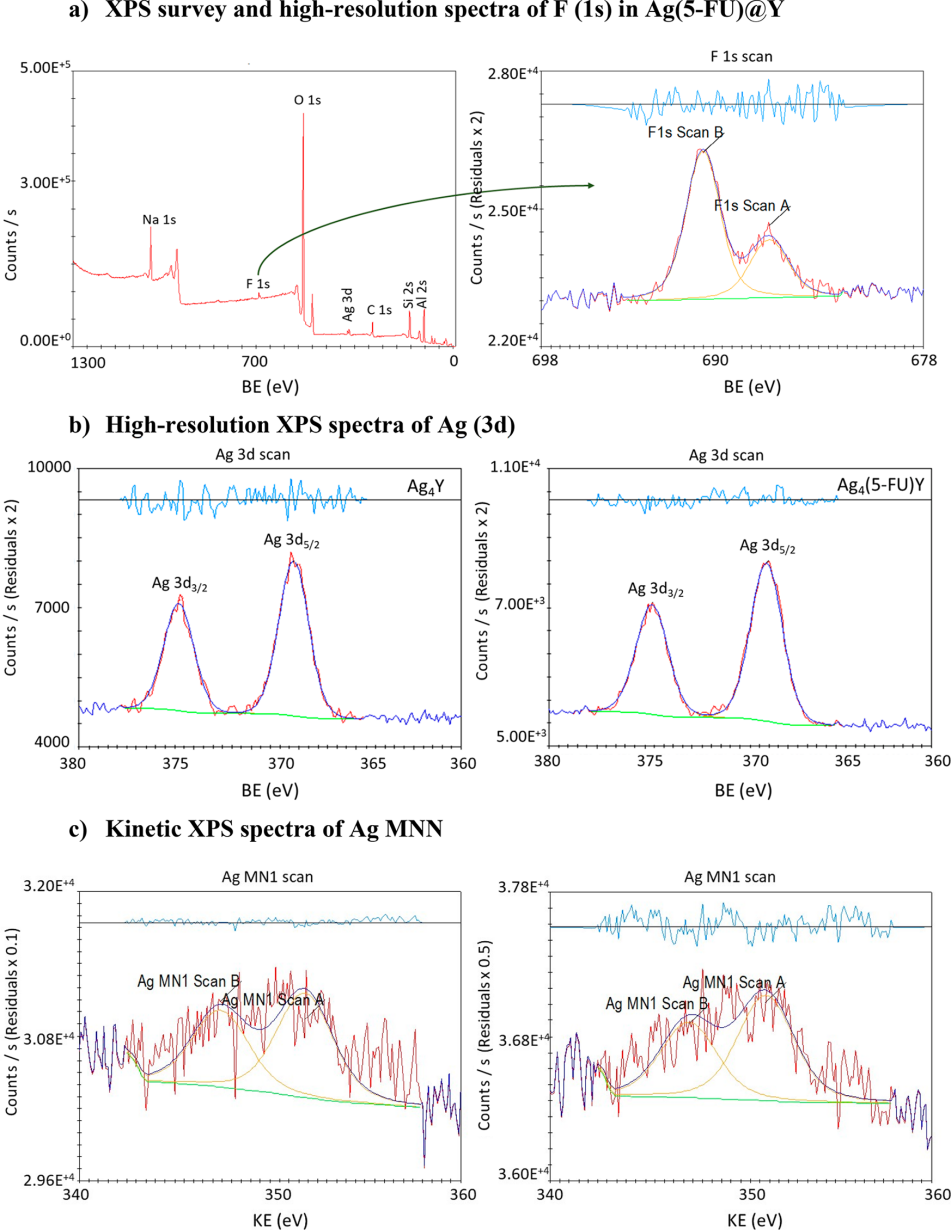
X-ray photoelectron spectroscopy (XPS) spectra of Ag(5-FU)@Y: (a) XPS survey and high-resolution spectra of the F 1s region; (b) high XPS resolution of the Ag 3d region; (c) kinetic spectra of the Ag MNN region.

The amount of silver quantified in AgY (ICP-AES) was 4.2 wt %, while the 5-FU loading obtained by TGA analysis was 0.770 mmol for (5-FU)@Y and 0.292 mmol for Ag(5-FU)@Y. In addition, the ZDS samples were also analyzed by XPS (Figure 1).

The presence of oxygen, sodium, aluminum, and silicon from the zeolite was detected. The confirmation of fluorine (Figure 1a) and silver (Figure 1b) at the surfaces of the ZDS samples was obtained by their characteristic binding energies (BE). The similar BE values obtained for Ag(5-FU)@Y and AgY suggest that the 5-FU loading did not interfere with the Ag chemical state (Figure 1b). The calculation of the Auger parameter allows us to determine the oxidation state of silver^31,32^ where values were 717.0 eV for AgY and 716.8 eV for Ag(5-FU)@Y, corresponding to the ionic state of the silver.^33^

**Figure 2:**
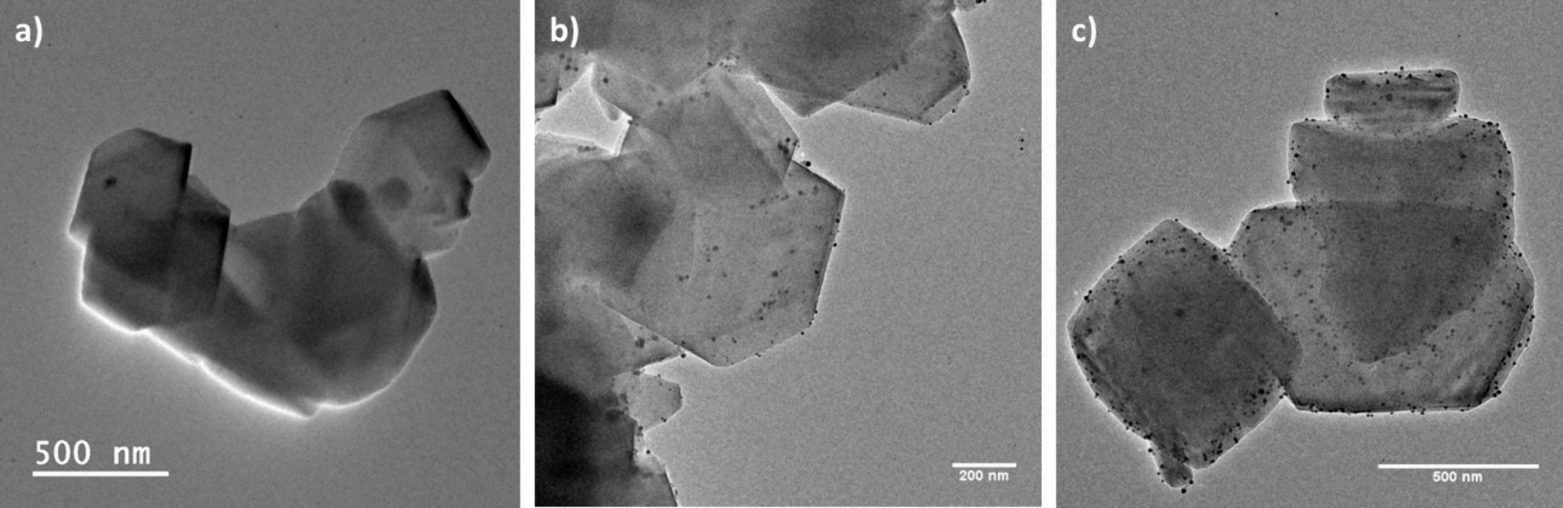
TEM images of (a) NaY,^35^ (b) AgY, and (c) Ag(5-FU)@Y.

The amount of silver on the surface corresponds to only about 35% of the total amount on the sample.^34^ TEM analysis was performed to confirm the presence of silver and whether the final particle sizes increased following the incorporation of both active species into the zeolite structure (ZDS) (Figure 2).

The results indicate that AgY and Ag(5-FU)@Y exhibit the characteristic morphology of zeolite crystalline particles, characterized by hexagonal crystals and uniform aggregates of intergrown zeolite nanocrystals. Furthermore, they maintain the original size of pristine NaY zeolite.^35^

To understand how the presence of silver affects the release of 5-FU, the 5-FU release profile of Ag(5-FU)@Y was studied at pH 7.4 to simulate physiological conditions (Figure S1). Only about 33% of the total 5-FU amount was released after 6 h. The presence of a high silver content hinders the drug’s penetration into the structure, resulting in an accumulation of 5-FU on the sample surface and hence rapid 5-FU desorption.^34^ However, the low content of Ag allows higher 5-FU loading into the structure as confirmed by the TGA and with only 0.032 mmol of 5-FU detected on the surface by XPS. These observations suggest that the zeolite with a low content of Ag enables a more controlled release of the drug.^34^

### Machine Learning (ML) Approaches

Silver and 5-FU have already shown antitumoral effects in several types of cancers.^36−38^ The effect of the prepared ZDS samples and further combinations was studied in the A375 melanoma cell line. Topical treatment applications are suitable for addressing skin cancer, and zeolites are recognized as promising candidates for this specific type of application.^39,40^ Furthermore, the administration of this therapy offers increased flexibility in terms of dosing and formulation, allowing for greater adaptability in treatment approaches. The pristine NaY zeolite did not interfere with cell viability in the tested range of concentrations and throughout the entire period of cell exposure to the sample (Figure S2).

To maximize the role of 5-FU, two different combinations of the systems were made: [Ag(5-FU)@Y + (5-FU)@Y] and [AgY + (5-FU)@Y], and a preliminary evaluation was performed using a full factorial design. The results were analyzed through the response surface methodology (RSM) as displayed in Figure S3. In both combinations, the results indicate an interaction between the two studied factors: ratio and concentration. In addition, the results show that the optimal Ag/5-FU ratio correlates with a higher concentration of 5-FU, resulting in decreased cell viability.

**Figure 3:**
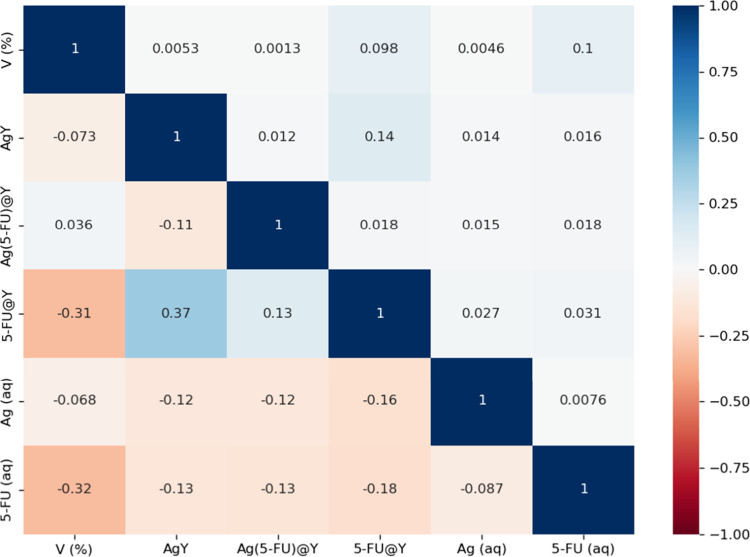
Heatmap representation of the correlation matrix concerning the variables at play: cell viability (V) and mass concentrations of Ag(aq), 5-FU(aq), AgY, (5-FU)@Y, and Ag(5-FU)@Y. The lower-left triangle represents Pearson’s correlation coefficient (*r*), while the upper-right triangle depicts its square (*r*^2^).

To help rationalize these results, two ML models were trained with the data from the individual cell viability assays, as described in the Experimental Section. Preliminary analysis of the data used in this work shows little correlation between cell viability and the mass composition of any of the five samples tested: Ag(aq), 5-FU(aq), AgY, (5-FU)@Y, and Ag(5-FU)@Y, as shown in Figure 3.

**Figure 4:**
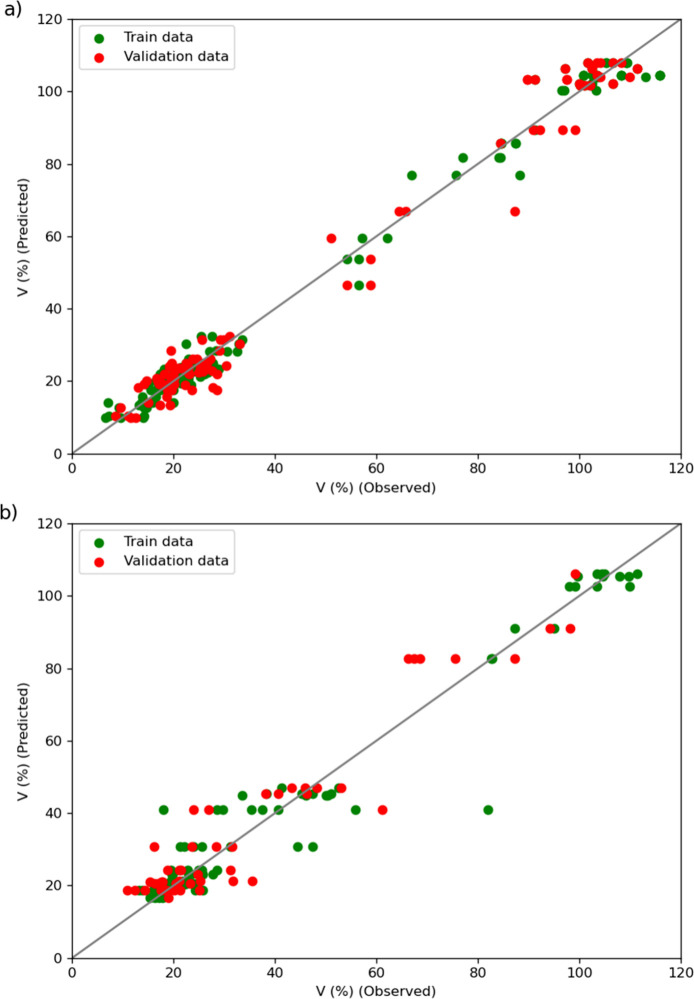
Fitness plot of the ANN model predicting cell viability (V) in aqueous solutions of Ag(aq) and or 5-FU(aq) (a) and of the ANN model predicting cell viability (V) of ZDF combinations of AgY, (5-FU)@Y, and Ag(5-FU)@Y (b).

Following the optimization of the model’s hyperparameters, model A was fitted using data from the assays using Ag and 5-FU in an aqueous solution. The fitness plot for this model shows excellent adherence to both the training and validation data (Figure 4a), with *r*^2^_train_ = 0.9901 and *r*^2^_val_ = 0.9781. The ANN model targeting cell viability as a function of the different ZDS compositions (model B) also shows good predictive capability with *r*^2^_train_ = 0.9425 and *r*^2^_val_ = 0.8972, as depicted in Figure 4b.

For the sake of simplicity, the discussion of the results from the virtual assays accepted using the two ANN models would be performed in terms of the fraction of the cell population affected by each component of the mixture. Henceforward is referred to simply as fraction affected, *f*_a_, which is defined as 1 – *V*, where *V* is the predicted cell viability, as a fraction of the initial cells.

### Dose–Response Curves

**Figure 5:**
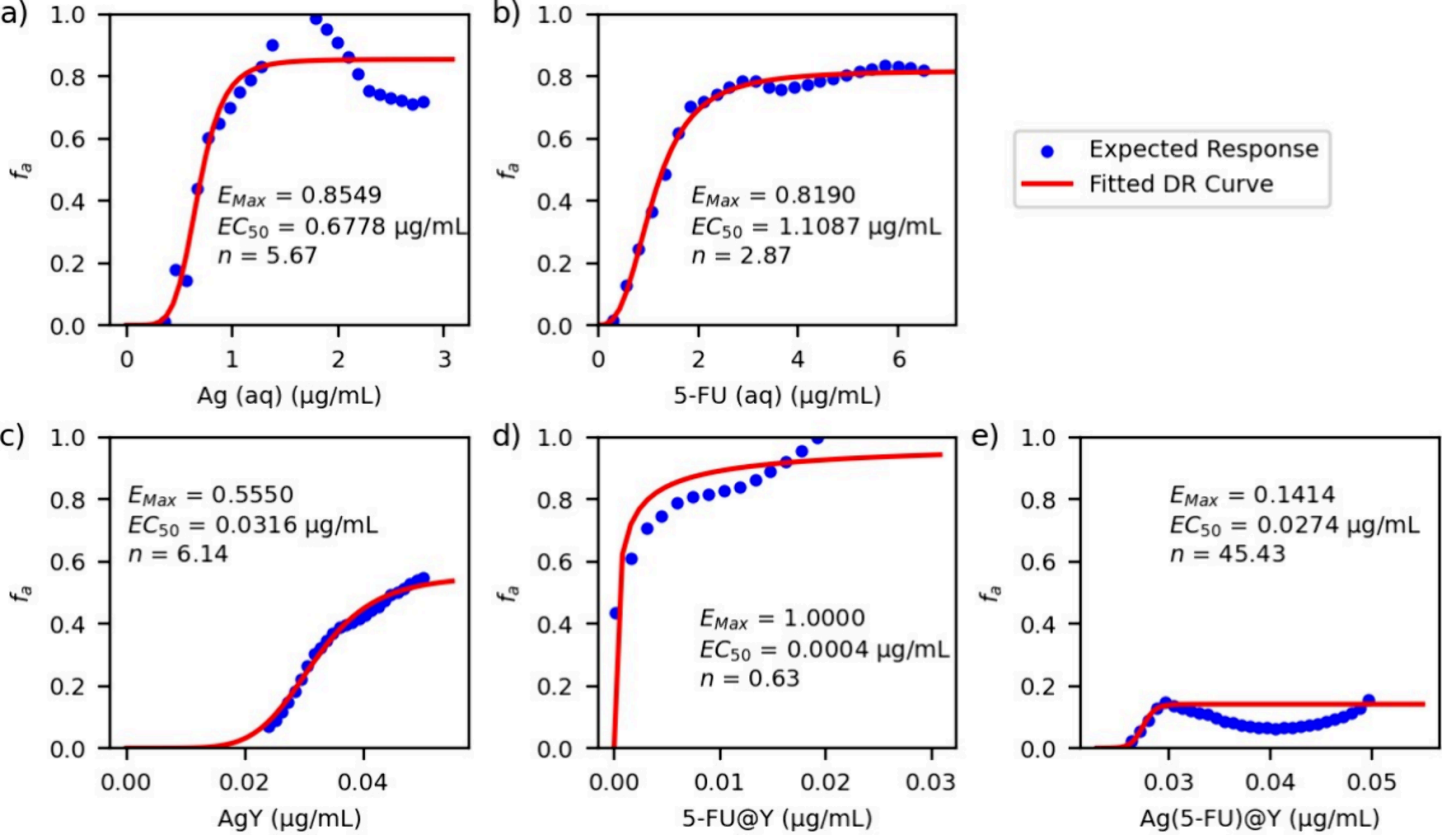
Dose–response data for Ag(aq) (a), 5-FU(aq) (b), AgY (c), (5-FU)@Y (d), and Ag(5-FU)@Y (e), retrieved from simulated data from the ANN models (blue dots). For each component, the adjusted curves derived from eq 1 are colored red, and the respective optimized parameters are given. In the case of Ag(5-FU)@Y (e), the dose–response data cannot be properly fitted using the model translated by eq 1, where *f*_a_ is nondimensional.

Model A was used to predict *f*_a_ for a grid of varying concentrations of Ag and 5-FU combined in aqueous solutions as well as *f*_a_ for pure Ag(aq) and pure 5-FU(aq) in concentrations ranging from 0.0 to 2.8 μg/mL (Ag) and from 0.0 to 6.5 μg/mL (5-FU). The fitted dose–response curves of Ag(aq) and 5-FU(aq) are depicted in Figures 5a and 5b, respectively, together with the data points retrieved from model A. These data were generated by feeding the model with varying concentrations of Ag(aq) while keeping the concentration of 5-FU(aq) at zero (for the Ag(aq) curve) and vice versa for the 5-FU(aq) dose–response curve. The fitted parameters for the former curve (Figure 5a) are *E*_max_ = 0.8549, EC_50_ = 0.6778 μg/mL, and *n* = 5.67, while those for 5-FU(aq) (Figure 5b) are *E*_max_ = 0.8190, EC_50_ = 1.1087 μg/mL, and *n* = 2.87. These results are in agreement with the experimental data depicted in Figure S4.

The dose–response curves for the solid phase (AgY, (5-FU)@Y, Ag(5-FU)@Y) show a very distinct behavior from their active species homologous to aqueous, as depicted in Figures 5c, 5d, and 5e, respectively. The data used for these dose–response curves were retrieved from the predictions of model B for single-component combinations of AgY, (5-FU)@Y, and Ag(5-FU)@Y, with doses varying up to 0.06, 0.03, and 0.05 μg/mL, respectively. Among the three ZDS tested, the dose–response behavior of AgY resembles the model depicted in eq 1 the most, with *E*_max_ = 0.5550, EC_50_ = 0.0316 μg/mL, and *n* = 6.14. Compared with its free aqueous phase analogue, incorporation of Ag into the zeolite framework decreases EC_50_ by over 1 order of magnitude, at the expense of not being able to achieve the *E*_max_ observed for Ag(aq). In the aqueous combinations, AgNO_3_ solutions were prepared with the same available concentrations on the ZDS samples. However, it is known that silver ions in contact with light can be reduced to metallic silver, Ag^0^.^25,41^

In contrast and as confirmed by the XPS analysis, silver is a cation in the ZDS samples (Figure 1). In addition, the composition of a complex medium with the presence of chloride anions, proteins, and amino acids can hinder the bioavailability of the silver ions.^42^ In the case of the ZDS samples, the silver ions seem to be stabilized by the strong electric fields within the framework, resulting in a strong attraction between Ag^+^ and the zeolite framework and consequently a very slow release.^10,34^ The study of Matsumura et al.^43^ proposed that the antibacterial activity of silver-containing zeolites is related to the release of Ag^+^ to a greater extent upon direct contact with the bacteria cell. According to the findings of Monteiro et al.,^9^ silver-loaded zeolite A exhibits its anticancer activity through the direct delivery of silver ions to the cells, resulting in increased oxidative stress caused by reactive oxygen species (ROS). This study also demonstrated lower cell viability in cells treated with the zeolite samples compared with the same mass concentration of silver nitrate. While the precise mechanism by which silver zeolites exert their anticancer activity remains unclear, it is evident that there are notable disparities in the chemical state and availability of silver between silver solutions and the silver found in ZDS samples. This disparity emphasizes the limitations of making a direct comparison between the two.

On the other hand, the cytotoxicity of 5-FU containing ZDS strongly depends on the zeolite. As depicted in Figure 5d, the cytotoxicity dose–response curve expected for (5-FU)@Y (from the predictions of ANN model B) also follows the model translated by eq 1, with optimized parameters *E*_max_ = 1.0000, EC_50_ = 0.0004 μg/mL, and *n* = 0.63. These values represent a huge decrease in EC_50_, compared to the free 5-FU in solution, while maintaining the maximum effect.

From Figure S1, only 33% of encapsulated 5-FU was released from the micropores over 6 h. These results seem to be in accordance with the dose–response behavior inferred from the available data using ANN model B. As shown in Figure 5e, the buffered suspensions of Ag(5-FU)@Y deviate from the typical sigmoid-like behavior for doses above 0.03 μg/mL. For the low-dose regime (dose ≤0.03 μg/mL), the optimized dose–response parameters are *E*_max_ = 0.1414, EC_50_ = 0.0274 μg/mL, and *n* = 45.43. As noted for the other ZDS systems, these values represent a huge decrease of EC_50_ relative to either Ag or 5-FU in aqueous medium, although at the expense of diminishing the *E*_max_. In the high-dosage regime (dose >0.03 μg/mL), the Ag(5-FU)@Y system does not present the typical sigmoid plateau, but its activity varies significantly, with a secondary activity maximum at about 0.05 μg/mL. Moreover, the maximum activity (fraction affected, *f*_a_) is close to 0.20, which is comparable only with the maximum activity observed for AgY, suggesting that the simultaneous incorporation of Ag and 5-FU into the zeolite may suppress the availability of 5-FU, yielding a cytotoxic profile closer to that of AgY, in line with the release profile of 5-FU. As a result of this study, IC_50_ for the different ZDS samples (solid phase), 5-FU, and Ag^+^ aqueous solutions (liquid phase) were determined: IC_50_ = 0.0453 μg/mL (AgY), IC_50_ = 0.0004 μg/mL (5-FU@Y), IC_50_ = 0.7200 μg/mL (Ag(aq)), and IC_50_ = 1.2966 μg/mL (5-FU(aq)).

### Prospective Synergistic/Antagonistic Effects with Combinations

Expanding on the results obtained for single-component preparations, the response of ANN model A was further explored over a grid of different concentrations of Ag and 5-FU. Simultaneously, the response of ANN model B was explored over a grid of different concentrations of AgY and (5-FU)@Y, constraining the concentration of Ag(5-FU)@Y to zero. What is more, for each point on the grid, the predicted response of the Ag/5-FU (or AgY/(5-FU)@Y) combination was compared with the sum of the response of the individual components alone, which served as a surrogate indicator of any antagonistic and/or synergistic behavior.

**Figure 6:**
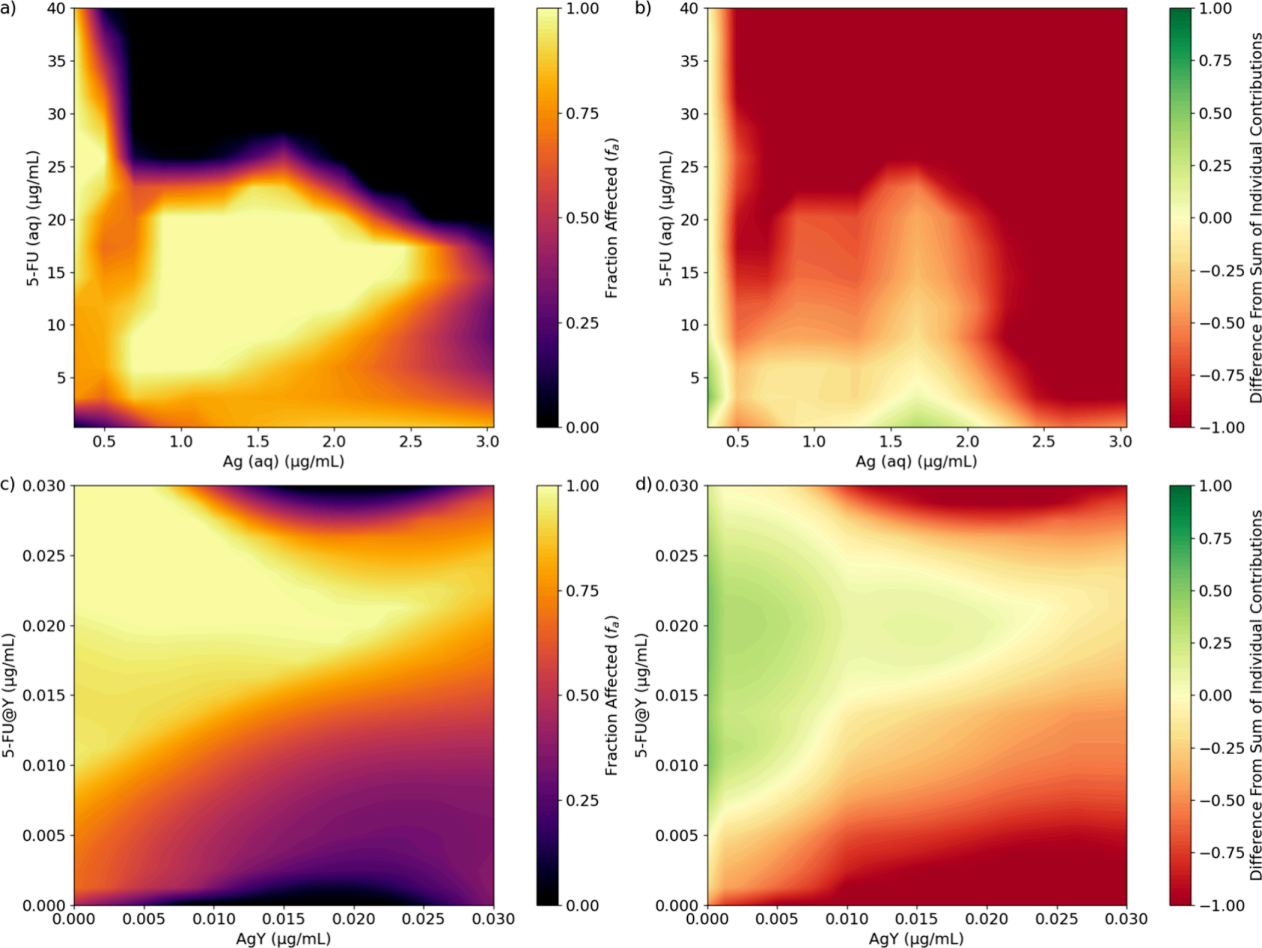
Dose–response diagrams of Ag/5-FU combinations in aqueous media (a) retrieved from the response of ANN model A and the remainder of the expected effect after subtracting the predicted effect of the individual components (Ag(aq) and 5-FU(aq)) (b). Dose–response diagrams of AgY/(5-FU)@Y combinations (c) retrieved from the response of ANN model B and the remainder of the expected effect after subtracting the predicted effect of the individual components (AgY and (5-FU)@Y) (d).

The results shown in Figure 6a depict the predicted activity (fraction affected) of aqueous Ag/5-FU combinations, derived from the response of ANN model A. The scan over this grid highlights the model’s inability to extrapolate the training data to the region for which the mass concentrations of Ag(aq) and 5-FU(aq) are simultaneously greater than 0.7 and 25 μg/mL, respectively. The model predicts a region of high activity for a one-component solution of 5-FU with a concentration of >5 μg/mL (Figure 5b). However, the activity of 5-FU(aq) in the region between 5 and 15 μg/mL appears to be hindered by the presence of small quantities of Ag, and maximum efficacy is only reattained for concentrations of Ag(aq) greater than 0.7 μg/mL. By subtracting the expected activity of the individual components (at the same concentration) from that of the Ag/5-FU mixture, one can identify that the combination of the two components is at best neutral and even antagonistic in some combinations, shown by the reddish hue in the lower left quadrant of Figure 6b.

Two possible exceptions to this observation were identified in Figure 6b and correspond to the following conditions: (a) concentration of 5-FU(aq) approximately equal to 5 μg/mL and minute amounts of Ag(aq) and (b) concentration of Ag(aq) between 1.5 and 2.0 μg/mL and minute quantities of 5-FU(aq). It should be noted that these exceptions take place near the IC_50_ of 5-FU(aq) (a) or Ag(aq) (b) and that these positive outcomes may be due to the numerical precision of the model in these regions of high variation.

The dose–response surface of AgY/(5-FU)@Y combinations retrieved from the response of ANN model B to a grid of varying concentrations of AgY and (5-FU)@Y is presented in Figure 6c. As mentioned above, the region of high (5-FU)@Y concentration and moderate AgY concentration is tainted by the model’s inability to extrapolate its training data onto that region. This artifact, however, covers a much smaller region of the surface plot than what is presented in Figure 6a for the Ag/5-FU combinations. Indeed, the most prominent feature of the data depicted in Figure 6c is the large triangular-shaped region denoting a range of AgY/(5-FU)@Y combinations for which the fraction affected is greater than 0.8. Moreover, after subtracting the response predicted for the individual AgY and (5-FU)@Y there is still a positive result, depicted in Figure 6d, suggesting a synergetic effect between AgY and (5-FU)@Y.

Our results suggest that this synergistic effect is more noticeable for AgY/(5-FU)@Y combinations with moderate doses of 5-FU@Y and small amounts of AgY, resulting in optimal AgY/(5-FU)@Y mass ratios of approximately 1:2 to 1:5. Noteworthy, the experimental assays with these ratios confirm the results obtained by the models, where 24.8% and 21.0% of cell viability were found for 1:2 to 1:5 ratios, respectively. Therefore, these studies confirm that both species Ag and 5-FU are potentiated in the solid state by their incorporation into the zeolite structure than in the liquid phase.

Incorporating 5-FU and Ag^+^ into the zeolite structure can provide several therapeutic benefits. The drug combination is already a common practice in cancer therapy, which improves anticancer activity and reduces the chance of developing resistance. Thus, having one formulation with two active agents will simplify administration to the patient. Particularly in this delivery system, the presence of Ag^+^ enhances the activity of 5-FU. Because 5-FU has severe side effects, the combination will allow a reduction of the dose of 5-FU, improving safety. These advancements in drug treatment can contribute to improving treatment outcomes and enhance the quality of life for individuals undergoing chemotherapy.

### Bacterial Assays

Increasing evidence suggests that microbes can influence the potential efficacy of small-drug chemotherapeutics. In addition, some bacteria have been identified as having the potential to induce various infections, including skin infections, thereby posing a risk to human health.^22^

In the case of drug efficacy, Lehouritis et al.^5^ reported that local bacteria can affect the efficacy of some drugs by showing the effect of *E. coli* (Gram-negative) and *Listeria welshimeri* (Gram-positive) in the presence of 30 drug chemotherapeutics. For example, bacteria decreased the cytotoxicity of doxorubicin, vidarabine, and gemcitabine, whereas the cytotoxicity of tegafur and fludarabine phosphate, two antimetabolites, increased. Otherwise, the cytotoxicity of 5-fluorouracil was unaffected in the presence of these bacteria.^5^ However, LaCourse et al.^45^ showed that 5-FU was affected by the presence of bacteria, and its activity was modified by intratumoral microbiota after 5-FU exposure, having the potential to deplete 5-FU levels, reducing local drug efficacy in colorectal cancer.

To understand the potential of silver to protect 5-FU in the ZDS samples, different bacterial assays were performed. The Gram-negative bacteria *E. coli* and the Gram-positive bacteria *S. aureus* were used as susceptible indicator strains to evaluate the antimicrobial potential of AgY. These microorganisms were selected because these strains are commonly responsible for skin infections and are also found in the microbiome of various cancers, thereby posing a risk to human health.^22^ Additionally, they serve as models for both Gram-positive and Gram-negative bacteria.^22,45^ Because these bacteria have important structural differences, their sensitivity to antibiotics varies, and thus, it is important to test any antimicrobial agent in both types of bacteria.

As expected, the pristine zeolite NaY has no antibacterial effects because the zeolites have been described as inert and devoid of antimicrobial properties.^10,24,46^ Other studies have already shown the potential of silver-loaded *faujasite* zeolites for both *E. coli* and *S. aureus*.^35,42,47,48^ Increasing concentrations of samples were tested, and the resulting MIC values were determined for each of the pairs of bacterial strain/samples (Table 1).

**Table 1:** MIC Values (mg/mL) for the Samples Tested against the Panel of the Tested Microorganisms

	NaY (mg/mL)		AgY (mg/mL)	
	24 h	48 h	24 h	48 h
*S. aureus*	>4	>4	0.5	1.0
*E. coli*	>4	>4	0.5	0.5

As the results show, the AgY sample has a significantly lower MIC compared with the initial pristine NaY zeolite, reinforcing the role of silver as an antimicrobial agent. The obtained MIC values suggest that *S. aureus* is less susceptible to the system compared to *E. coli*. Hanim et al.’s findings also reveal a disparity in the MIC values for *E. coli* and *S. aureus* upon exposure to zeolite-loaded silver samples.^48^ The authors suggest that this difference may be attributed to differences in the cell wall structure, as *E. coli* is a Gram-negative bacterium while *S. aureus* is Gram-positive. Consequently, the thicker cell wall of *S. aureus* could potentially hinder the penetration of Ag ions into the cell membrane.^48,49^

The ANN models successfully predicted that the most effective combinations of AgY/(5-FU)@Y mass ratios were 1:2 and 1:5. To evaluate the antimicrobial activity against *S. aureus* and *E. coli*, different mass ratios of AgY/(5-FU)@Y (1:1 and 1:5) and Ag(5-FU)@Y/(5-FU)@Y (1:5 and 5:1) were tested using the agar well diffusion tests and compared with (5-FU)@Y, and NaY. In the agar well diffusion test, a lower concentration of the ZDS combinations (50 μg/mL) was employed. This concentration was selected to maintain consistency with the conditions employed in cytotoxicity assessments for cancer cell assays.^34^

At a concentration of 50 μg/mL, the ratio of 1:5 exhibited antibacterial activity against *S. aureus*, while no inhibition was observed for *E. coli* with all samples. As anticipated, NaY did not display any antibacterial activity at this concentration, and the same was true for AgY in both bacteria. Interestingly, the ratio 1:1 AgY/(5-FU)@Y also showed antibacterial activity against *S. aureus*, indicating that the other ratio predicted by the ANN models, 1:2, likely exhibits the same behavior as the 1:1 and 1:5 ratios against the same bacterium (Figure S5).

Our previous work has demonstrated that 5-FU possesses antimicrobial properties.^34^ However, in the context of cancer resistance associated with bacteria, it is crucial to validate these findings using a strain known to affect the activity of 5-FU.^44^ Future studies can be conducted using such a model to gain further insights into the role of silver (Ag) in these models, investigating whether it can act as a protective, antibacterial, and antineoplastic agent, potentially leading to a synergistic effect when combined with 5-FU.

## Conclusions

This study aimed to explore the potential of zeolite-based formulations combining 5-FU and silver for cancer therapy using machine learning (ML) tools. To determine the most effective formulation, two different ANN models were utilized. A novel protocol was developed for virtual cell viability assays. The results indicate that the introduction of 5-FU and Ag^+^ in the zeolite samples enhances their efficiency. However, it was also observed that a dual system might not necessarily yield a better response, possibly due to the potential impairment of 5-FU release in the presence of silver. ML models suggested two optimal ratios, 1:2 and 1:5 of AgY/(5-FU)@Y, both of which were subsequently validated by experimental data showing low cell viability values as well as in the antimicrobial activity. This approach holds promise for addressing cutaneous lesions resulting from skin cancer and microbial infections that commonly occur in vulnerable and injured skin areas. Furthermore, this work demonstrates that ANN can effectively learn the drug-delivery behavior of a ZDS. Future work will address the expansion of this modeling technique to other drug-delivery systems based on porous materials such as different zeolite structures, metal–organic frameworks, and covalent–organic frameworks. Indeed, having demonstrated the ability of ANN algorithms to learn the rather complex behavior of multiload ZDS, future work may use this method even further, allowing the ML-based modeling of the *in vivo* drug delivery process.

## Supporting Information Available

Release profile of 5-FU from Ag(5-FU)@Y under physiological conditions, biocompatibility of zeolite NaY with A375 cells, response surface plots presenting the effect of the studied variables with ZDS combinations, dose–response data derived from experimental cell viability assays, antimicrobial activity assays with various ZDS samples against *S. aureus*, date for machine learning analysis (PDF)
