# Correction to “Machine Learning-Assisted Optimization of Drug Combinations in Zeolite-Based Delivery Systems for Melanoma Therapy”

**DOI:** 10.1021/acsami.4c04116

**Published:** 2024-03-19

**Authors:** Ana Raquel Bertão, Filipe Teixeira, Viktoriya Ivasiv, Pier Parpot, Cristina Almeida-Aguiar, António M. Fonseca, Manuel Bañobre-López, Fátima Baltazar, Isabel C. Neves

In our original article, we have identified a discrepancy in the Supporting Information (SI). Specifically, there are certain data points that were intended for inclusion but were inadvertently omitted, specifically the Python notebooks for all data analysis used in the paper. We modified the SI from pages S-8 to S-50, where the Python notebooks for all data analysis used in the paper are added. In the same revised SI, from pages S-6 to S-7, we introduce Table S2. I want to emphasize that these omitted data points in the SI do not affect the analysis or conclusions drawn in the paper; rather, they serve to complement and enhance the context of our research findings. The additional data provide supplementary insights that may be beneficial to interested readers and researchers in our field.

## Supporting Information Available

Release profile of 5-FU from Ag(5-FU)@Y under physiological conditions, biocompatibility of zeolite NaY with A375 cells, response surface plots presenting the effect of the studied variables with ZDS combinations, dose–response data derived from experimental cell viability assays, antimicrobial activity assays with various ZDS samples against *S. aureus*, date for machine learning analysis, and Python notebooks for all data analysis used in the paper (PDF)

