# Supplementary material for: Machine Learning-Assisted Optimization of Drug Combinations in Zeolite-Based Delivery Systems for Melanoma Therapy
Source: ACS Appl Mater Interfaces. 2024 Jan 25;16(5):5696–707. doi: 10.1021/acsami.3c18224 (PMC10859889; doi:10.1021/acsami.3c18224)
Supplement: Supplementary file 1 — am3c18224_si_001.pdf [file am3c18224_si_001.pdf]

Supporting Information

Machine learning-assisted optimization of drug combinations in zeolite-based delivery systems for melanoma therapy

Ana Raquel Bertão,[†,‡,§,||,*] *Filipe Teixeira,*[†] *Viktoriya Ivasiv,*[†] *Pier Parpot,*[†,⊥] *Cristina Almeida-Aguiar,*[#] *António M. Fonseca,*[†,⊥] *Manuel Bañobre-López,*[||] *Fátima Baltazar,*[‡,§] *Isabel C. Neves,*[†,⊥,**]

[†]CQUM, Centre of Chemistry, University of Minho, Campus de Gualtar, 4710-057 Braga, Portugal

[‡]Life and Health Sciences Research Institute (ICVS), School of Medicine, University of Minho, 4710-057 Braga, Portugal.

[§]ICVS/3B's - PT Government Associate Laboratory, University of Minho, 4710-057 Braga/Guimarães, Portugal.

[||]Advanced (magnetic) Theranostic Nanostructures Lab, Nanomedicine Group International Iberian Nanotechnology Laboratory (INL), Av. Mestre José Veiga, 4715-330 Braga, Portugal

[⊥]CEB - Centre of Biological Engineering, University of Minho, 4710-057 Braga, Portugal

[#]CBMA - Centre of Molecular and Environmental Biology, University of Minho, 4710-057 Braga, Portugal.

[*]*ana.bertao@inl.int*

[**]*ineves@quimica.uminho.pt*

Tel.: +351253601552 and Fax:+351253604382

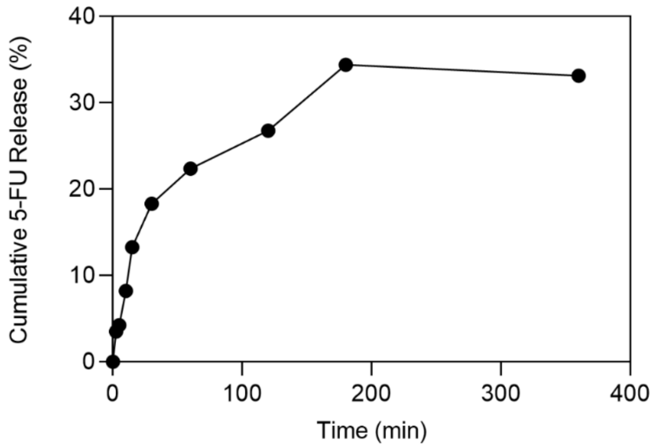

Figure S1. Release profile of 5-FU from Ag(5-FU)@Y in PBS solution at pH = 7.4 over 360 min.

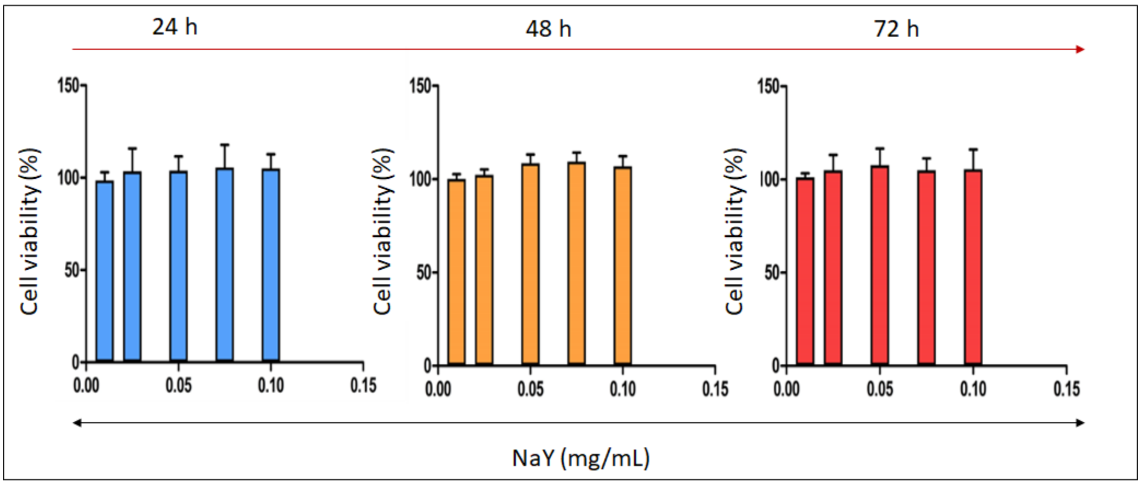

Figure S2. Cell viability of A375 cells measured by SRB assay after cell incubation with increasing concentrations of NaY throughout 72 h. The resulting percentages (calculated as the mean percentage ± SD of viability) were calculated in relation to the control, meaning that cells cultured in the presence of 0 mg/mL of NaY were considered as 100% of viability.

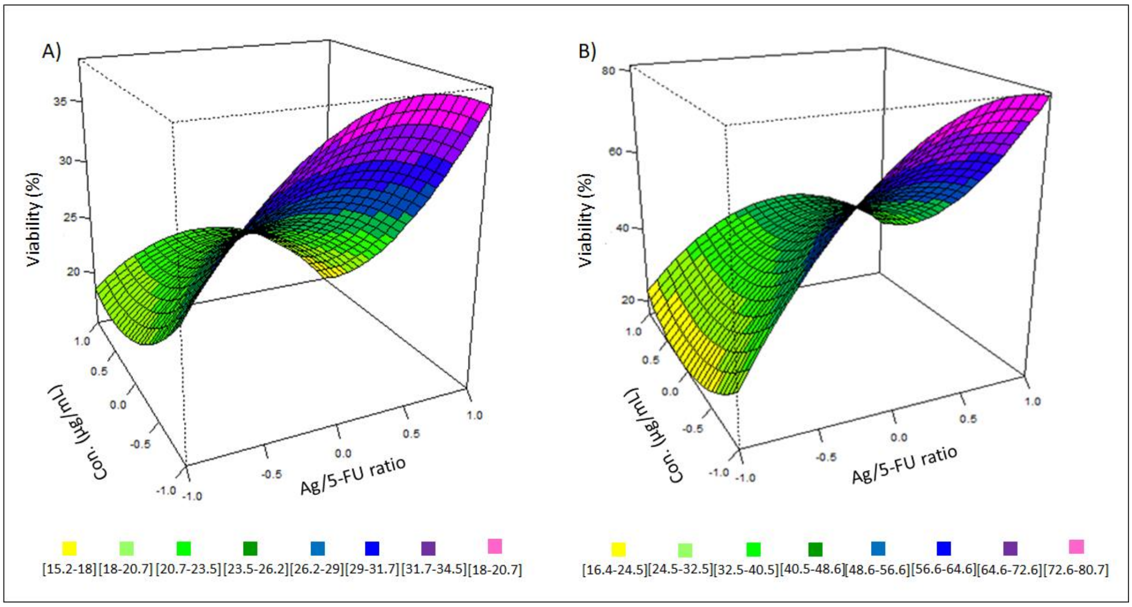

Figure S3. 3D Response Surface Plots (RSP): Effect of the two variables studied with ZDS combinations: A) (5-FU)@Y and AgY, and B) (5-FU)@Y and Ag(5-FU)@Y. Cell viability of A375 cells measured by SRB assay after cell incubation with increasing concentrations of ZDS combinations (10, 25 and 50 µmL) throughout 72 h. The resulting percentages (calculated as the mean percentage ± SD of viability) were calculated in relation to the control, meaning that cells cultured in the presence of 0 mg/mL of ZDS combinations were considered as 100% of viability.

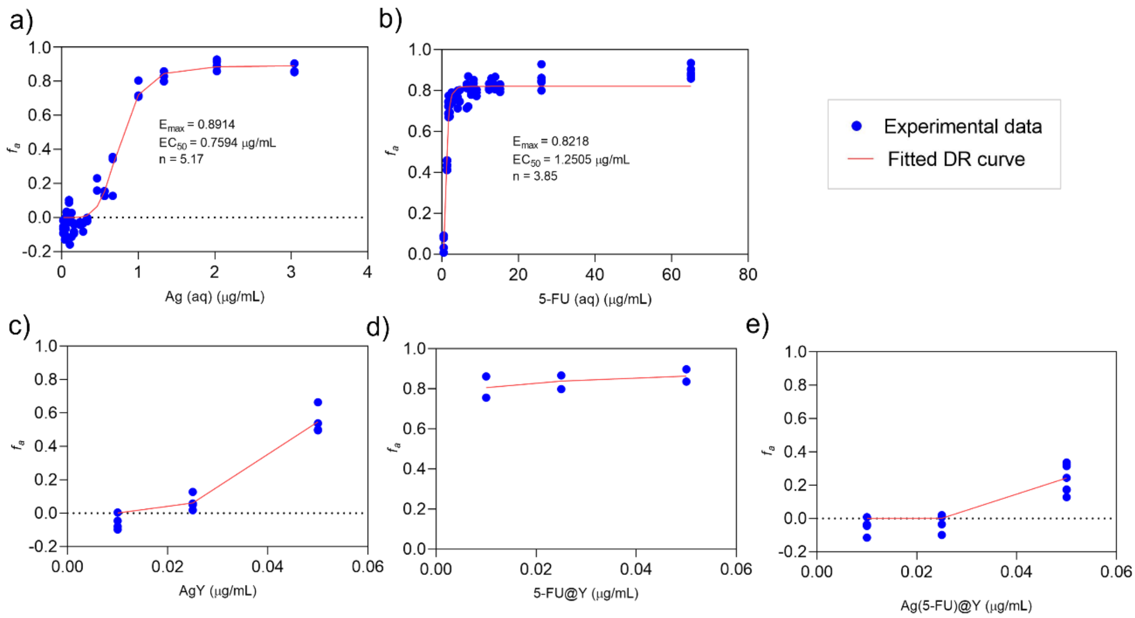

Figure S4. Dose-response data for Ag (aq) (a), 5-FU (aq) (b), AgY (c), (5-FU)@Y (d), and Ag(5-FU)@Y (e), retrieved from experimental data of the cell viability assays (blue dots). For each component, the adjusted curves derived from Eq. (1) are depicted in red, and the respective optimized parameters are given. In the case of Ag(5-FU)@Y (e), the dose-response data cannot be properly fitted using the model translated by Eq. (1), where f_a is non-dimensional.

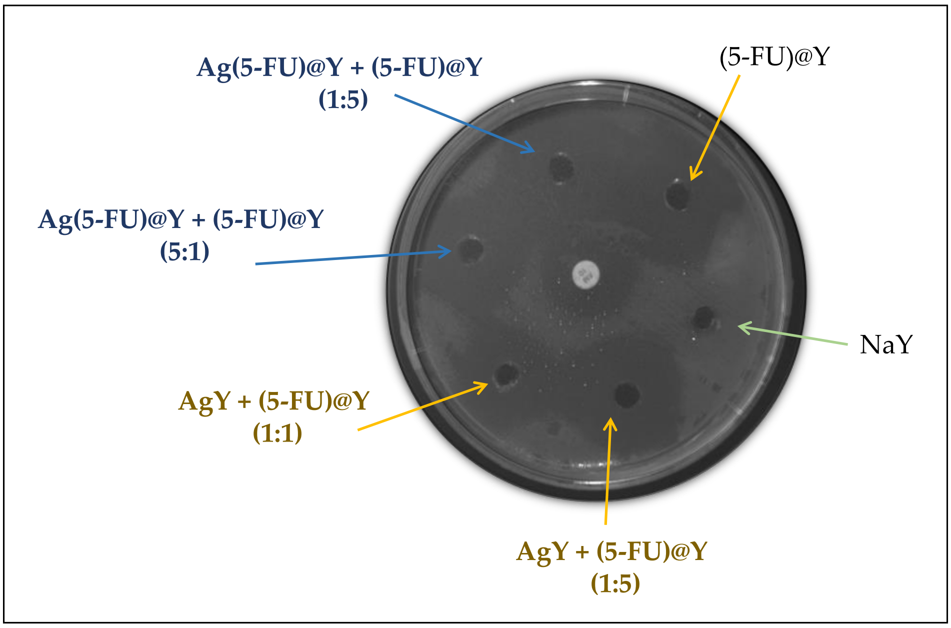

Figure S5. Antimicrobial activity assay with NaY, (5-FU)@Y and different ratios of AgY/(5-FU)@Y (1:1 and 1:5) and Ag(5-FU)@Y/(5-FU)@Y (1:5 and 5:1) against *S. aureus*, after 24 h of incubation using agar well diffusion tests.

Table S1. Data of 127 points of the different combinations used for all machine-learning calculations.

Sample	Replica	ZDS] (mg/l	Ag (s)	5-FU (s)	Ag (aq)	5-FU (aq)	ll viab. (V,
S2	S2_1	0.01	0.5	20	0	0	38.07319
S2	S2_2	0.01	0.5	20	0	0	38.37209
S2	S2_3	0.01	0.5	20	0	0	51.09706
S2	S2_4	0.01	0.5	20	0	0	45.2993
S2	S2_5	0.01	0.5	20	0	0	46.29392
S2	S2_6	0.01	0.5	20	0	0	40.62431
S2	S2_7	0.01	0.5	20	0	0	47.45327
S3	S3_1	0.025	1.2	50	0	0	21.70433
S3	S3_2	0.025	1.2	50	0	0	22.46113
S3	S3_3	0.025	1.2	50	0	0	31.72183
S3	S3_4	0.025	1.2	50	0	0	25.23818
S3	S3_5	0.025	1.2	50	0	0	35.52429
S3	S3_6	0.025	1.2	50	0	0	21.06511
S3	S3_7	0.025	1.2	50	0	0	19.44088
S4	S4_1	0.05	2.6	95	0	0	22.07078
S4	S4_2	0.05	2.6	95	0	0	17.07914
S4	S4_3	0.05	2.6	95	0	0	18.6971
S5	S5_1	0	0	0	0.05393	0	106.5755
S5	S5_2	0	0	0	0.05393	0	102.4308
S5	S5_3	0	0	0	0.05393	0	100.0103
S6	S6_1	0	0	0	0.129432	0	102.4308
S6	S6_2	0	0	0	0.129432	0	97.23734
S6	S6_3	0	0	0	0.129432	0	111.3267
S7	S7_1	0	0	0	0.280436	0	108.288
S7	S7_2	0	0	0	0.280436	0	102.862
S7	S7_3	0	0	0	0.280436	0	103.4181
S8	S8_1	0	0	0	0	2.6016	20.89591
S8	S8_2	0	0	0	0	2.6016	21.44436
S8	S8_3	0	0	0	0	2.6016	21.63057
S8	S8_4	0	0	0	0	2.6016	25.27245
S9	S9_1	0	0	0	0	6.504	17.32069
S9	S9_2	0	0	0	0	6.504	16.82466
S9	S9_3	0	0	0	0	6.504	18.72562
S9	S9_4	0	0	0	0	6.504	28.55283
S10	S10_1	0	0	0	0	12.3576	16.57957
S10	S10_2	0	0	0	0	12.3576	18.71776
S10	S10_3	0	0	0	0	12.3576	19.57052
S10	S10_4	0	0	0	0	12.3576	19.39566
S15	S15_1	0.01	0.2	20	0	0	24.96664
S15	S15_2	0.01	0.2	20	0	0	31.21067
S15	S15_3	0.01	0.2	20	0	0	19.48961
S15	S15_4	0.01	0.2	20	0	0	22.7946
S15	S15_5	0.01	0.2	20	0	0	25.54853
S15	S15_6	0.01	0.2	20	0	0	21.03182
S15	S15_7	0.01	0.2	20	0	0	28.62168
S15	S15_8	0.01	0.2	20	0	0	18.74417
S15	S15_9	0.01	0.2	20	0	0	21.51834
S16	S16_1	0.025	0.4	59	0	0	20.51639
S16	S16_2	0.025	0.4	59	0	0	23.98204
S16	S16_3	0.025	0.4	59	0	0	16.24805
S16	S16_4	0.025	0.4	59	0	0	19.76961
S16	S16_5	0.025	0.4	59	0	0	23.31722
S16	S16_6	0.025	0.4	59	0	0	20.45659
S16	S16_7	0.025	0.4	59	0	0	22.59837
S16	S16_8	0.025	0.4	59	0	0	16.80163
S16	S16_9	0.025	0.4	59	0	0	15.92716
S17	S17_1	0.05	0.9	117	0	0	17.09699
S17	S17_2	0.05	0.9	117	0	0	18.93617
S17	S17_3	0.05	0.9	117	0	0	17.85008
S17	S17_4	0.05	0.9	117	0	0	15.3132
S17	S17_5	0.05	0.9	117	0	0	16.33752
S18	S18_1	0	0	0	0.021572	0	109.2449
S18	S18_2	0	0	0	0.021572	0	105.3198
S18	S18_3	0	0	0	0.021572	0	106.6325
S18	S18_4	0	0	0	0.021572	0	101.6081
S19	S19_1	0	0	0	0.043144	0	109.9254
S19	S19_2	0	0	0	0.043144	0	112.9944
S19	S19_3	0	0	0	0.043144	0	103.7245
S20	S20_1	0	0	0	0.097074	0	89.79629
S20	S20_2	0	0	0	0.097074	0	97.51043
S20	S20_3	0	0	0	0.097074	0	91.29044
S21	S21_1	0	0	0	0	2.6016	20.89591
S21	S21_2	0	0	0	0	2.6016	21.44436
S21	S21_3	0	0	0	0	2.6016	21.63057
S21	S21_4	0	0	0	0	2.6016	25.27245
S22	S22_1	0	0	0	0	7.67472	17.68891
S22	S22_2	0	0	0	0	7.67472	21.96021
S22	S22_3	0	0	0	0	7.67472	21.48342
S23	S23_1	0	0	0	0	15.21936	16.84178
S23	S23_2	0	0	0	0	15.21936	19.2336
S23	S23_3	0	0	0	0	15.21936	20.63733
S28	S28_1	0.01	0.9	14	0	0	48.2408
S28	S28_2	0.01	0.9	14	0	0	52.44901
S28	S28_3	0.01	0.9	14	0	0	52.99368
S28	S28_4	0.01	0.9	14	0	0	41.30865
S28	S28_5	0.01	0.9	14	0	0	43.30622
S28	S28_6	0.01	0.9	14	0	0	45.9605
S29	S29_1	0.025	2.2	36	0	0	19.73121
S29	S29_2	0.025	2.2	36	0	0	24.81919
S29	S29_3	0.025	2.2	36	0	0	19.67274
S29	S29_4	0.025	2.2	36	0	0	23.44059
S29	S29_5	0.025	2.2	36	0	0	27.78633
S29	S29_6	0.025	2.2	36	0	0	25.70701
S29	S29_7	0.025	2.2	36	0	0	22.92896
S30	S30_1	0.05	4.3	70	0	0	15.38571
S30	S30_2	0.05	4.3	70	0	0	20.97164
S30	S30_3	0.05	4.3	70	0	0	17.60892
S30	S30_4	0.05	4.3	70	0	0	17.87638
S31	S31_1	0	0	0	0.097074	0	89.79629
S31	S31_2	0	0	0	0.097074	0	97.51043
S31	S31_3	0	0	0	0.097074	0	91.29044
S32	S32_1	0	0	0	0.237292	0	103.4068
S32	S32_2	0	0	0	0.237292	0	104.1669
S32	S32_3	0	0	0	0.237292	0	103.4213
S32	S32_4	0	0	0	0.237292	0	102.7409
S33	S33_1	0	0	0	0.463798	0	84.15623
S33	S33_2	0	0	0	0.463798	0	76.92095
S33	S33_3	0	0	0	0.463798	0	84.43196
S34	S34_1	0	0	0	0	1.82112	32.94151
S34	S34_2	0	0	0	0	1.82112	22.43988
S34	S34_3	0	0	0	0	1.82112	32.84749
S35	S35_1	0	0	0	0	4.68288	19.72606
S35	S35_2	0	0	0	0	4.68288	19.60206
S35	S35_3	0	0	0	0	4.68288	19.9016
S35	S35_4	0	0	0	0	4.68288	25.30924
S36	S36_1	0	0	0	0	9.1056	20.85085
S36	S36_2	0	0	0	0	9.1056	22.59013
S36	S36_3	0	0	0	0	9.1056	19.47611
S41	S41_1	0.01	0.6	13	0	0	29.77974
S41	S41_2	0.01	0.6	13	0	0	40.55235
S41	S41_3	0.01	0.6	13	0	0	37.50903
S41	S41_4	0.01	0.6	13	0	0	35.35654
S42	S42_1	0.025	1.5	32	0	0	23.96843
S42	S42_2	0.025	1.5	32	0	0	31.55219
S42	S42_3	0.025	1.5	32	0	0	22.04884
S42	S42_4	0.025	1.5	32	0	0	23.52789
S43	S43_1	0.05	3.1	64	0	0	21.30236
S43	S43_2	0.05	3.1	64	0	0	25.12577
S43	S43_3	0.05	3.1	64	0	0	25.72494
S43	S43_4	0.05	3.1	64	0	0	24.30439
S44	S44_1	0	0	0	0.064716	0	96.53453
S44	S44_2	0	0	0	0.064716	0	103.3172
S44	S44_3	0	0	0	0.064716	0	97.03519
S45	S45_1	0	0	0	0.16179	0	109.4278
S45	S45_2	0	0	0	0.16179	0	108.2108
S45	S45_3	0	0	0	0.16179	0	103.3907
S45	S45_4	0	0	0	0.16179	0	104.0567
S46	S46_1	0	0	0	0.334366	0	102.1901
S46	S46_2	0	0	0	0.334366	0	100.1093
S46	S46_3	0	0	0	0.334366	0	100.9902
S47	S47_1	0	0	0	0	1.69104	25.39728
S47	S47_2	0	0	0	0	1.69104	27.59896
S47	S47_3	0	0	0	0	1.69104	31.09787
S48	S48_1	0	0	0	0	4.16256	19.93634
S48	S48_2	0	0	0	0	4.16256	23.16161
S48	S48_3	0	0	0	0	4.16256	28.63305
S49	S49_1	0	0	0	0	8.32512	16.40894
S49	S49_2	0	0	0	0	8.32512	20.8757
S49	S49_3	0	0	0	0	8.32512	14.67378
S54	S54_1	0.01	0.2	21	0	0	23.86387
S54	S54_2	0.01	0.2	21	0	0	26.89721
S54	S54_3	0.01	0.2	21	0	0	18.00318
S54	S54_4	0.01	0.2	21	0	0	28.47159
S55	S55_1	0.025	0.5	53	0	0	16.10093
S55	S55_2	0.025	0.5	53	0	0	21.29693
S55	S55_3	0.025	0.5	53	0	0	25.6213
S55	S55_4	0.025	0.5	53	0	0	28.44571
S56	S56_1	0.05	1	107	0	0	10.82979
S56	S56_2	0.05	1	107	0	0	17.44426
S56	S56_3	0.05	1	107	0	0	18.95256
S56	S56_4	0.05	1	107	0	0	18.93651
S56	S56_5	0.05	1	107	0	0	13.22063
S57	S57_1	0	0	0	0.021572	0	109.2449
S57	S57_2	0	0	0	0.021572	0	105.3198
S57	S57_3	0	0	0	0.021572	0	106.6325
S57	S57_4	0	0	0	0.021572	0	101.6081
S58	S58_1	0	0	0	0.05393	0	106.5755
S58	S58_2	0	0	0	0.05393	0	102.4308
S58	S58_3	0	0	0	0.05393	0	100.0103
S59	S59_1	0	0	0	0.10786	0	102.5969
S59	S59_2	0	0	0	0.10786	0	100.7623
S59	S59_3	0	0	0	0.10786	0	115.838
S60	S60_1	0	0	0	0	2.73168	21.00762
S60	S60_2	0	0	0	0	2.73168	25.8173
S60	S60_3	0	0	0	0	2.73168	23.74485
S61	S61_1	0	0	0	0	6.89424	27.69823
S61	S61_2	0	0	0	0	6.89424	17.00218
S61	S61_3	0	0	0	0	6.89424	12.96836
S62	S62_1	0	0	0	0	13.91856	19.28312
S62	S62_2	0	0	0	0	13.91856	17.24515
S62	S62_3	0	0	0	0	13.91856	13.22432
S67	S67_1	0.01	1	4	0	0	81.89399
S67	S67_2	0.01	1	4	0	0	55.90233
S67	S67_3	0.01	1	4	0	0	61.04987
S68	S68_1	0.025	2.6	11	0	0	44.41531
S68	S68_2	0.025	2.6	11	0	0	47.48194
S68	S68_3	0.025	2.6	11	0	0	31.21565
S69	S69_1	0.05	5.2	21	0	0	13.85307
S69	S69_2	0.05	5.2	21	0	0	18.47874
S69	S69_3	0.05	5.2	21	0	0	20.06175
S69	S69_4	0.05	5.2	21	0	0	12.35371
S69	S69_5	0.05	5.2	21	0	0	16.02174
S69	S69_6	0.05	5.2	21	0	0	14.35455
S69	S69_7	0.05	5.2	21	0	0	18.55135
S70	S70_1	0	0	0	0.10786	0	102.5969
S70	S70_2	0	0	0	0.10786	0	100.7623
S70	S70_3	0	0	0	0.10786	0	115.838
S71	S71_1	0	0	0	0.280436	0	108.288
S71	S71_2	0	0	0	0.280436	0	102.862
S71	S71_3	0	0	0	0.280436	0	103.4181
S72	S72_1	0	0	0	0.560872	0	84.63347
S72	S72_2	0	0	0	0.560872	0	84.46498
S72	S72_3	0	0	0	0.560872	0	87.33696
S73	S73_1	0	0	0	0	0.52032	96.75527
S73	S73_2	0	0	0	0	0.52032	90.89839
S73	S73_3	0	0	0	0	0.52032	91.18758
S73	S73_4	0	0	0	0	0.52032	92.1466
S73	S73_5	0	0	0	0	0.52032	99.22387
S74	S74_1	0	0	0	0	1.43088	54.14262
S74	S74_2	0	0	0	0	1.43088	58.82573
S74	S74_3	0	0	0	0	1.43088	56.5365
S75	S75_1	0	0	0	0	2.73168	21.00762
S75	S75_2	0	0	0	0	2.73168	25.8173
S75	S75_3	0	0	0	0	2.73168	23.74485
S80	S80_1	0.01	1.2	0	0	0	109.7181
S80	S80_2	0.01	1.2	0	0	0	104.5802
S80	S80_3	0.01	1.2	0	0	0	107.9902
S80	S80_4	0.01	1.2	0	0	0	99.59881
S81	S81_1	0.025	3.1	0	0	0	87.2928
S81	S81_2	0.025	3.1	0	0	0	98.12686
S81	S81_3	0.025	3.1	0	0	0	94.17714
S81	S81_4	0.025	3.1	0	0	0	94.93982
S82	S82_1	0.05	6.2	0	0	0	50.35896
S82	S82_2	0.05	6.2	0	0	0	46.11157
S82	S82_3	0.05	6.2	0	0	0	33.56451
S82	S82_4	0.05	6.2	0	0	0	49.98901
S83	S83_1	0	0	0	0.129432	0	102.4308
S83	S83_2	0	0	0	0.129432	0	97.23734
S83	S83_3	0	0	0	0.129432	0	111.3267
S84	S84_1	0	0	0	0.334366	0	102.1901
S84	S84_2	0	0	0	0.334366	0	100.1093
S84	S84_3	0	0	0	0.334366	0	100.9902
S85	S85_1	0	0	0	0.668732	0	64.45822
S85	S85_2	0	0	0	0.668732	0	65.68187
S85	S85_3	0	0	0	0.668732	0	87.2548
S89	S89_1	0	0	26	0	0	13.82154
S89	S89_2	0	0	26	0	0	24.357
S90	S90_1	0	0	64	0	0	13.30963
S90	S90_2	0	0	64	0	0	20.13629
S91	S91_1	0	0	130	0	0	10.20974
S91	S91_2	0	0	130	0	0	16.39921
S92	S92_1	0.01	1	12	0	0	104.4425
S92	S92_2	0.01	1	12	0	0	99.10308
S92	S92_3	0.01	1	12	0	0	111.4255
S92	S92_4	0.01	1	12	0	0	104.8987
S92	S92_5	0.01	1	12	0	0	103.5092
S93	S93_1	0.025	2.6	30	0	0	109.8482
S93	S93_2	0.025	2.6	30	0	0	103.4686
S93	S93_3	0.025	2.6	30	0	0	97.97369
S93	S93_4	0.025	2.6	30	0	0	99.12798
S94	S94_1	0.05	5.2	61	0	0	75.50658
S94	S94_2	0.05	5.2	61	0	0	66.3333
S94	S94_3	0.05	5.2	61	0	0	87.17682
S94	S94_4	0.05	5.2	61	0	0	82.55857
S94	S94_5	0.05	5.2	61	0	0	68.56013
S94	S94_6	0.05	5.2	61	0	0	67.44339
S94	S94_7	0.05	5.2	61	0	0	82.79046
S95	S95_1	0	0	0	0.10786	0	102.5969
S95	S95_2	0	0	0	0.10786	0	100.7623
S95	S95_3	0	0	0	0.10786	0	115.838
S96	S96_1	0	0	0	0.280436	0	108.288
S96	S96_2	0	0	0	0.280436	0	102.862
S96	S96_3	0	0	0	0.280436	0	103.4181
S97	S97_1	0	0	0	0.560872	0	84.63347
S97	S97_2	0	0	0	0.560872	0	87.33696
S97	S97_3	0	0	0	0.560872	0	84.46498
S101	S101_1	0	0	0	0.05393	2.6016	21.52117
S101	S101_2	0	0	0	0.05393	2.6016	25.00614
S101	S101_3	0	0	0	0.05393	2.6016	25.9714
S101	S101_4	0	0	0	0.05393	2.6016	27.51644
S102	S102_1	0	0	0	0.129432	6.504	17.6284
S102	S102_2	0	0	0	0.129432	6.504	22.89364
S102	S102_3	0	0	0	0.129432	6.504	25.71389
S103	S103_1	0	0	0	0.280436	12.3576	15.51057
S103	S103_2	0	0	0	0.280436	12.3576	19.89683
S103	S103_3	0	0	0	0.280436	12.3576	23.65384
S104	S104_1	0	0	0	0.021572	2.6016	19.24198
S104	S104_2	0	0	0	0.021572	2.6016	26.01326
S104	S104_3	0	0	0	0.021572	2.6016	26.85428
S104	S104_4	0	0	0	0.021572	2.6016	30.31223
S105	S105_1	0	0	0	0.043144	7.67472	19.64538
S105	S105_2	0	0	0	0.043144	7.67472	21.37067
S105	S105_3	0	0	0	0.043144	7.67472	22.88132
S105	S105_4	0	0	0	0.043144	7.67472	25.9714
S106	S106_1	0	0	0	0.097074	15.21936	15.04667
S106	S106_2	0	0	0	0.097074	15.21936	18.57038
S106	S106_3	0	0	0	0.097074	15.21936	23.50669
S106	S106_4	0	0	0	0.097074	15.21936	22.25594
S107	S107_1	0	0	0	0.097074	1.82112	23.82053
S107	S107_2	0	0	0	0.097074	1.82112	22.89364
S107	S107_3	0	0	0	0.097074	1.82112	27.18536
S107	S107_4	0	0	0	0.097074	1.82112	24.5735
S108	S108_1	0	0	0	0.237292	4.68288	17.99146
S108	S108_2	0	0	0	0.237292	4.68288	21.71457
S108	S108_3	0	0	0	0.237292	4.68288	24.8678
S108	S108_4	0	0	0	0.237292	4.68288	26.70713
S109	S109_1	0	0	0	0.463798	9.1056	18.15281
S109	S109_2	0	0	0	0.463798	9.1056	20.92852
S109	S109_3	0	0	0	0.463798	9.1056	24.13206

S109	S109_4	0	0	0	0.463798	9.1056	28.80397
S110	S110_1	0	0	0	0.064716	1.69104	29.18597
S110	S110_2	0	0	0	0.064716	1.69104	25.64921
S110	S110_3	0	0	0	0.064716	1.69104	29.92092
S110	S110_4	0	0	0	0.064716	1.69104	33.51928
S111	S111_1	0	0	0	0.16179	4.16256	22.35482
S111	S111_2	0	0	0	0.16179	4.16256	25.13358
S111	S111_3	0	0	0	0.16179	4.16256	23.16935
S112	S112_1	0	0	0	0.334366	8.32512	16.61797
S112	S112_2	0	0	0	0.334366	8.32512	18.7915
S112	S112_3	0	0	0	0.334366	8.32512	22.92287
S112	S112_4	0	0	0	0.334366	8.32512	20.15523
S113	S113_1	0	0	0	0.021572	2.73168	19.64892
S113	S113_2	0	0	0	0.021572	2.73168	24.00202
S113	S113_3	0	0	0	0.021572	2.73168	25.61242
S114	S114_1	0	0	0	0.05393	6.89424	19.12635
S114	S114_2	0	0	0	0.05393	6.89424	25.17859
S114	S114_3	0	0	0	0.05393	6.89424	27.9262
S114	S114_4	0	0	0	0.05393	6.89424	24.27853
S115	S115_1	0	0	0	0.10786	13.91856	17.32345
S115	S115_2	0	0	0	0.10786	13.91856	20.00168
S115	S115_3	0	0	0	0.10786	13.91856	26.04545
S115	S115_4	0	0	0	0.10786	13.91856	18.21489
S116	S116_1	0	0	0	0.10786	0.52032	88.20909
S116	S116_2	0	0	0	0.10786	0.52032	75.7112
S116	S116_3	0	0	0	0.10786	0.52032	66.89304
S117	S117_1	0	0	0	0.280436	1.43088	62.16062
S117	S117_2	0	0	0	0.280436	1.43088	57.11404
S117	S117_3	0	0	0	0.280436	1.43088	51.12203
S118	S118_1	0	0	0	0.560872	2.73168	26.57308
S118	S118_2	0	0	0	0.560872	2.73168	22.42205
S118	S118_3	0	0	0	0.560872	2.73168	22.42205
S118	S118_4	0	0	0	0.560872	2.73168	27.6882
S119	S119_1	0	0	0	0	1.3008	54.14262
S119	S119_2	0	0	0	0	1.3008	58.82573
S119	S119_3	0	0	0	0	1.3008	56.5365
S120	S120_1	0	0	0	0	2.08128	32.59956
S120	S120_2	0	0	0	0	2.08128	30.52275
S120	S120_3	0	0	0	0	2.08128	27.01916
S121	S121_1	0	0	0	0	13.008	13.87921
S121	S121_2	0	0	0	0	13.008	16.49897
S121	S121_3	0	0	0	0	13.008	18.58858
S122	S122_1	0	0	0	0	26.016	7.00495
S122	S122_2	0	0	0	0	26.016	13.73458
S122	S122_3	0	0	0	0	26.016	15.778
S122	S122_4	0	0	0	0	26.016	15.07222
S122	S122_5	0	0	0	0	26.016	19.90423
S123	S123_1	0	0	0	0	65.04	6.49962
S123	S123_2	0	0	0	0	65.04	9.50855
S123	S123_3	0	0	0	0	65.04	12.56554
S123	S123_4	0	0	0	0	65.04	11.58547
S123	S123_5	0	0	0	0	65.04	14.02873
S124	S124_1	0	0	0	1.003098	0	19.53105
S124	S124_2	0	0	0	1.003098	0	29.00905
S124	S124_3	0	0	0	1.003098	0	28.38444
S125	S125_1	0	0	0	1.337464	0	20.04724
S125	S125_2	0	0	0	1.337464	0	14.11593
S125	S125_3	0	0	0	1.337464	0	17.08159
S126	S126_1	0	0	0	2.027768	0	7.2377
S126	S126_2	0	0	0	2.027768	0	8.4713
S126	S126_3	0	0	0	2.027768	0	14.09058
S126	S126_4	0	0	0	2.027768	0	11.03565
S127	S127_1	0	0	0	3.041652	0	14.1096
S127	S127_2	0	0	0	3.041652	0	9.5134
S127	S127_3	0	0	0	3.041652	0	14.66571
S127	S127_4	0	0	0	3.041652	0	9.19234

where Ag(aq) and 5-FU(aq) correspond to the liquid phase, and Ag(s) and 5-FU(s) correspond to the solid phase.
